# From Water to Bloodstream: Aeromonas dhakensis as an Emerging Cause of Catheter-Related Bloodstream Infection

**DOI:** 10.7759/cureus.115420

**Published:** 2026-08-29

**Authors:** Aditi Gupta, Vivek Hada, Manish Chaturvedy, Rajesh Jhorawat, Vibhor Tak, Kumar S Abhishek, Kuntal Sinha

**Affiliations:** 1 Microbiology, All India Institute of Medical Sciences, Jodhpur, Jodhpur, IND; 2 Nephrology, All India Institute of Medical Sciences, Jodhpur, Jodhpur, IND

**Keywords:** aeromonas dhakensis, bloodstream infections (bsi), catheter-related bloodstream infection, haemodialysis, sepsis

## Abstract

Catheter-related bloodstream infections are a significant complication in haemodialysis patients. Water-associated Gram-negative organisms are increasingly recognised in this setting. We report a case of catheter-related bloodstream infection due to *Aeromonas dhakensis* in a 52-year-old woman on maintenance haemodialysis. The organism was identified using MALDI-TOF MS and showed in vitro susceptibility to ceftazidime. However, the clinical response to the antibiotic remained suboptimal until catheter removal. Repeat blood cultures after removal were sterile. This case highlights the emerging clinical relevance of *A. dhakensis* and the importance of correlating microbiological findings with clinical response.

## Introduction

Catheter-related bloodstream infection remains a frequent and clinically important complication in patients receiving maintenance haemodialysis. Although arteriovenous fistulas are preferred because of their lower risk of infection, long-term central venous catheters are often unavoidable. Such dependence increases the risk of bloodstream infection, particularly when there are lapses in catheter care and infection control practices [1].

Haemodialysis patients are repeatedly exposed to water systems, dialysis equipment, and disinfectants. Additionally, dialysis across multiple centres may increase the risk of contamination, as consistency in hand hygiene and catheter handling practices cannot be ensured across facilities. As a result, water-associated Gram-negative organisms are increasingly recognised as a cause of dialysis-related infections [2].

*Aeromonas dhakensis *is a water-associated Gram-negative organism that has emerged as a clinically relevant pathogen in recent years. Previously considered part of the *Aeromonas hydrophila* complex, it was reclassified as a distinct species in 2013 [3]. Infections due to *A. dhakensis* are often linked to environmental water exposure and may be under-recognised in routine practice [4]. We report a case of catheter-related bloodstream infection due to *A. dhakensis* in a haemodialysis patient.

## Case presentation

A 52-year-old woman presented to the dialysis unit for a haemodialysis session. During dialysis, she developed chills and rigours, leading to discontinuation of the session. Similar episodes had occurred a week prior during dialysis sessions at another centre. She also reported mild pain and tenderness at the right internal jugular vein catheter site.

She was admitted to the Nephrology department for further management. On examination, she was febrile but haemodynamically stable, with a blood pressure of 140/80 mmHg and a pulse rate of 85 bpm. Oxygen saturation was normal on room air. There was no erythema, warmth, or purulent discharge from the catheter site, and systemic examination was otherwise unremarkable. Laboratory investigations showed leukocytosis (16,384 cells/µL), a C-reactive protein level of 112 mg/L, and a procalcitonin level of 49.25 ng/mL. Paired blood cultures were obtained from the central venous catheter and a peripheral vein, and empirical intravenous ceftazidime 1 g twice daily, with a second dose administered post-dialysis, was started.

She had a five-year history of chronic kidney disease and was on maintenance haemodialysis. There was a history of arteriovenous fistula failure requiring multiple catheter insertions, and the patient had known diabetes mellitus and hypertension. She had completed treatment for pulmonary tuberculosis five years earlier and reported a history of pericarditis and myocardial infarction. Notably, she had undergone dialysis at multiple regional centres prior to referral.

Both blood culture sets flagged positive, with time to positivity of 9 hours 25 minutes for the catheter-drawn sample and 6 hours 27 minutes for the peripheral sample, with a differential time to positivity of >2 hours. Both sets were drawn simultaneously with comparable blood volumes. Thus, a diagnosis of catheter-related bloodstream infection was made. Culture showed moist colonies with a wide zone of beta-haemolysis on blood agar and non-lactose-fermenting colonies on MacConkey agar (Figure 1). The isolate was catalase- and oxidase-positive and identified as *Aeromonas dhakensis* by MALDI-TOF MS (Matrix-Assisted Laser Desorption/Ionization Time-of-Flight Mass Spectrometry, bioMérieux, France). Antimicrobial susceptibility testing was performed by disc diffusion and interpreted according to CLSI M45. The organism was susceptible to piperacillin-tazobactam, cefepime, ceftazidime, meropenem, imipenem, amikacin and ciprofloxacin. 

**Figure 1:**
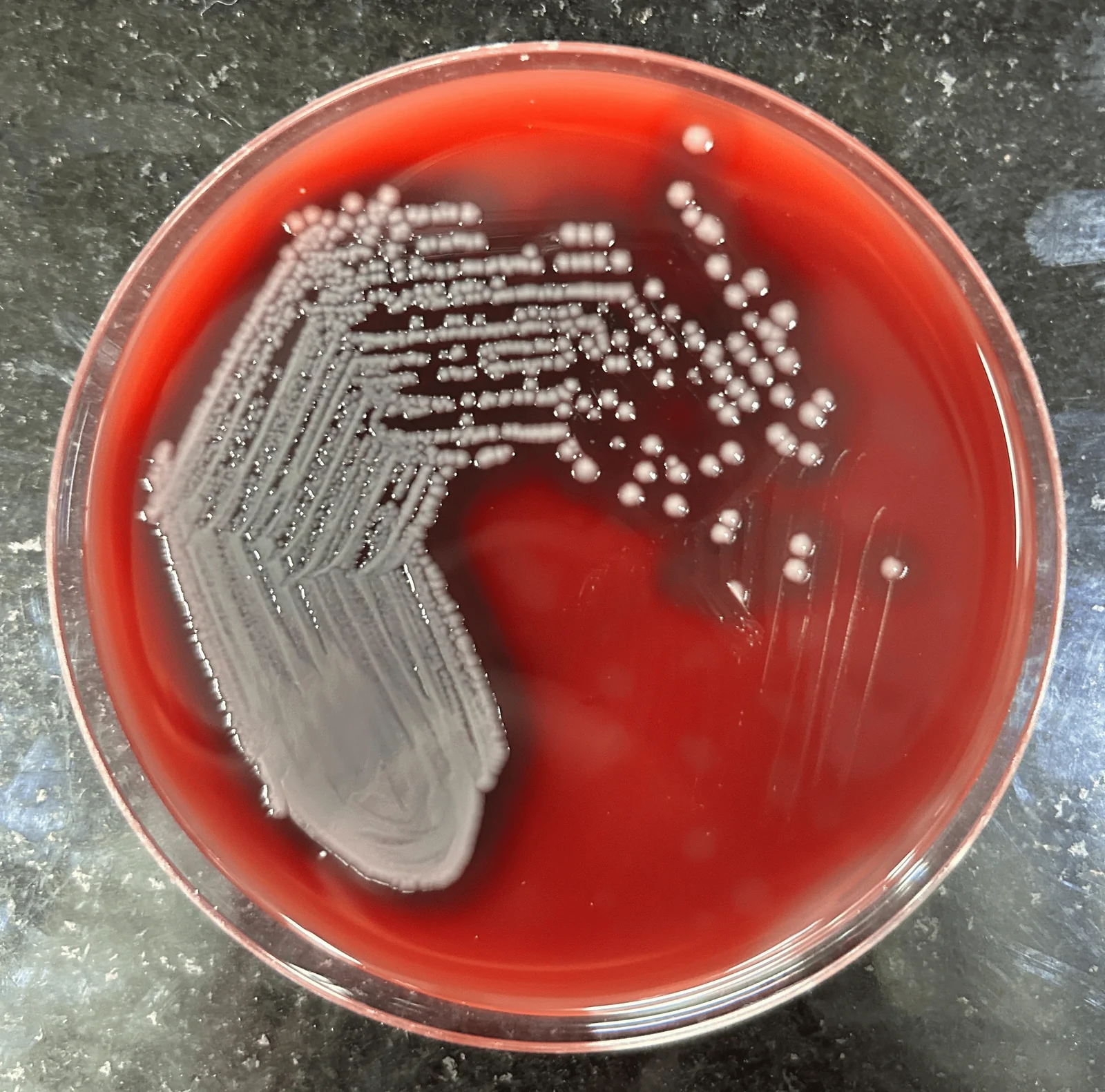
Growth on sheep blood agar showing smooth, moist, greyish colonies with a surrounding zone of β-haemolysis

Despite appropriate therapy, the patient remained febrile, and inflammatory markers showed only marginal decline over 72 hours. Urine and respiratory cultures were sterile, and no alternate focus was identified. A persistent catheter-related infection with probable biofilm was suspected, and the catheter was removed and replaced. Culture of the removed catheter tip grew *Aeromonas dhakensis* with a susceptibility profile similar to the blood isolate. Subsequently, intravenous ceftazidime was continued for three days after catheter removal, completing a total treatment duration of five days. 

Following catheter removal and replacement, the patient showed rapid clinical improvement. Leukocyte count decreased to 7,500 cells/µL, CRP to 24 mg/L, and procalcitonin to 4.38 ng/mL. Repeat blood cultures were sterile. The patient was discharged in stable condition with advice for outpatient follow-up. On routine follow-up during subsequent haemodialysis visits, no recurrent fever or bacteraemia was observed.

## Discussion

*Aeromonas dhakensis *has been increasingly recognised as a cause of serious human infections, including bacteraemia and necrotising fasciitis [5]. The organism was first described in 2002 after being isolated from stool samples of children with diarrhoeal illness in Dhaka, Bangladesh [6]. Its natural habitat is aquatic, and it is well established as a pathogen in aquaculture, where it causes disease in fish and other aquatic animals [7].

Over the past decade, the pathogenic potential of *A. dhakensis* has become clearer. Studies from Taiwan have reported higher fatality rates in this species than in other members of the *Aeromonas hydrophila* complex, emphasising its increased virulence [8]. Several published case reports and series have documented severe and fatal infections caused by *A. dhakensis* [5,9-11]. In India, only one other case of human infection due to this organism has been reported, and that case proved fatal [12]. The organism has been identified in Indian water bodies and aquaculture environments, suggesting a local environmental reservoir [13].

In the present case, the organism was identified as *A. dhakensis* by MALDI-TOF mass spectrometry. Accurate speciation is important, as this organism is often misidentified as *Aeromonas hydrophila* and may otherwise be overlooked in routine practice [8]. In settings where MALDI-TOF MS is unavailable, presumptive identification of *Aeromonas *species can be aided by conventional biochemical features, such as a positive oxidase reaction, glucose fermentation, nitrate reduction, and a triple sugar iron test showing an alkaline slant and acid butt. Growth of yellow, sucrose-fermenting colonies on thiosulphate citrate bile salt sucrose agar is an additional useful phenotypic marker, along with the distilled water motility test [14]. 

Although the isolate from this patient was reported as susceptible to ceftazidime in vitro, the agent used empirically produced a suboptimal clinical response, characterised by persistent fever and elevated inflammatory markers after 72 hours. Multiple factors could be responsible, including inadequate source control, inducible resistance mechanisms, and biofilm on the indwelling catheter. Antimicrobial resistance in *A. dhakensis* is an area of increasing concern. It* *carries chromosomally encoded class B, C, and D β-lactamases, including species-specific AmpC enzyme *blaAQU-1*, which can lead to treatment failure despite apparent susceptibility [15]. Isolates of *Aeromonas dhakensis* obtained from sewage water in India have also been shown to harbour colistin resistance mediated by the *mcr-1 *gene [16].

This case study has certain limitations. Molecular testing for resistance determinants was not performed, and biofilm formation on the catheter was not directly demonstrated. Environmental cultures at our haemodialysis unit were sterile, but as data from other dialysis facilities were unavailable, the route of acquisition could not be established.

In this patient, clinical improvement was observed only after removal and replacement of the central venous catheter, which was accompanied by a decline in inflammatory markers.This clinical course, supported by a positive catheter tip culture for the same organism with the same susceptibility profile, is consistent with catheter-related infection. Indirect introduction through contaminated water sources, dialysis equipment, or catheter handling remains a plausible explanation, although a definitive source could not be identified. Isolation of *A. dhakensis* from blood cultures should therefore prompt careful clinical monitoring, awareness of potential inducible resistance, and early consideration of catheter removal. Strict adherence to infection control practices within dialysis units remains essential to reduce the risk of such infections.

## Conclusions

*Aeromonas dhakensis* is an emerging cause of catheter-related bloodstream infection in haemodialysis patients. Exposure to water systems or dialysis equipment across multiple centres may represent a contributing risk factor. Accurate laboratory speciation is essential, as this organism can be misidentified and its in vitro susceptibility may not predict clinical response. Early catheter removal and replacement should be considered when clinical improvement is not achieved despite appropriate antimicrobial therapy. This case reinforces the importance of correlating microbiological findings with clinical course and maintaining strict infection control practices within dialysis units. 
